# Draft Genome Sequence of an Escherichia coli Sequence Type 410 Isolate Harboring *bla*_NDM-5_ Genes with *gyrA* and *parE* Mutations, Isolated from Urine Culture in China

**DOI:** 10.1128/MRA.01088-19

**Published:** 2019-12-12

**Authors:** Junwei Huang, Shanshan Ma, Qian Yu, Miao Fu, Lingli Shao

**Affiliations:** a Department of Clinical Laboratory, Jinhua Central Hospital, Jinhua, Zhejiang, China; b Department of Clinical Laboratory, Beijing Northern Hospital of Weaponry Industry, Beijing, China; Indiana University, Bloomington

## Abstract

Escherichia coli sequence type 410 (ST410) is now an international high-risk clone and is responsible for a large number of clinical infections. Here, we report the draft genome sequence of an ST410 clinical isolate harboring multiple *bla*_NDM-5_ genes that was obtained from urine culture in China.

## ANNOUNCEMENT

Global dissemination of New Delhi metallo-β-lactamase (NDM)-producing bacteria poses a serious global public health threat, as the mortality associated with invasive infections caused by these species is high (1). Escherichia coli, a facultative anaerobic Gram-negative bacterium, is one of the major life-threatening pathogens that causes nosocomial infections (2). It is noteworthy that Escherichia coli sequence type 410 (ST410) has been identified as a potential pandemic clone causing a wide variety of clinical infections (3). We describe here the complete genome sequence of one E. coli ST410 strain, ECS5, harboring *bla*_NDM-5_ genes with *gyrA* and *parE* mutations. Urine (10 μl) from a 45-year-old man with a urinary tract infection who had been admitted to Jinhua Central Hospital (Zhejiang, China) was cultured on Columbia sheep blood agar at 35°C for 18 to 24 h. Representative colonies were subcultured on MacConkey agar overnight at 35°C to be used for species identification with the Vitek 2 automated system (bioMérieux, Marcy-l’Étoile, France) in the clinical microbiology laboratory of Jinhua Central Hospital in 2018.

Strain ECS5 was grown on Columbia blood agar prior to sequencing. Genomic DNA of strain ECS5 was extracted using a DNeasy blood and tissue kit (Qiagen, Germany) in accordance with the manufacturer’s instructions. Genomic DNA purity and concentration were evaluated by agarose gel electrophoresis and measured by using a Qubit fluorometer (Thermo Fisher).The DNA library was prepared using a Nextera XT DNA library preparation kit (Illumina, Inc., Cambridge, UK), and genomic DNA was sequenced on an Illumina HiSeq 4000 instrument with a 150-bp paired-end approach at a depth of approximately 200×. As a quality control step, Illumina PCR adapter reads and low-quality reads from the paired ends were filtered by Trim Galore v.0.4.1 with default parameters (https://www.bioinformatics.babraham.ac.uk/projects/trim_galore/). All good-quality paired reads of the carbapenem-resistant E. coli strain ECS5 were assembled using the SPAdes genome assembler v.3.13.0 with default setting (http://cab.spbu.ru/software/spades/) (4), which yielded 87 contigs with an *N*_50_ value of 128,960 bp, and the length of the assembled genome was 4,864,484 bp. The overall GC content of this E. coli ST410 strain was 50.55%, and whole-genome sequence data analyses were performed using the following bioinformatics tools with default parameters: ResFinder v.3.1, MLST v.2.0, VirulenceFinder v.2.0, PlasmidFinder v.2.0, pMLST v.2.0, and FimTyper v.1.0 (available from the Center for Genomic Epidemiology (http://www.genomicepidemiology.org/).

Analysis of the draft genome sequences demonstrated that the carbapenem-resistant E. coli ECS5 strain belongs to multilocus sequence typing (MLST) sequence type 410 (ST410), whereas identification of plasmid replicons revealed that it carried four replicons (IncX3, IncFII, IncFIA, and IncFIB). In addition, Escherichia coli strain ST410 was assigned *in silico* to serotype 08:H9, and two potential virulence genes, namely *lpfA* (long polar fimbriae) and *iss* (increased serum survival), were detected. Using a 90% identity threshold, resistance genes to aminoglycosides (*aadA5*), β-lactams (*bla*_NDM-5_), macrolides (*mphA* and *mdfA*), sulfonamides (*sull*), tetracyclines (*tetA*), and trimethoprim (*dfrA17*) were identified. In addition, a double mutation in *gyrA* (Asp87Asn and Ser83Leu), as well as a single mutation in *parE* (Ser458Ala), were detected. Additional mutations in *parC* (Ser80Ile) were also identified. Carriage of these mutations may suggest a high resistance to fluoroquinolones in the investigated E. coli strain (5).

The presented genome sequences of carbapenemase-producing Escherichia coli strain ST410 could help global efforts to trace and provide further insight into the acquisitions of the carbapenemase gene *bla*_NDM-5_ of this successful lineage.

### Data availability.

The complete genome sequence of the E. coli ST410 isolate reported here has been deposited at DDBJ/ENA/GenBank under accession no. VMHS00000000. The raw data have been deposited in the SRA database under accession no. SRR9088960.
